# Animal models to study the neurological manifestations of the post-COVID-19 condition

**DOI:** 10.1038/s41684-023-01231-z

**Published:** 2023-08-24

**Authors:** Carla Usai, Lourdes Mateu, Christian Brander, Júlia Vergara-Alert, Joaquim Segalés

**Affiliations:** 1 Unitat Mixta d’Investigació IRTA-UAB en Sanitat Animal, Centre de Recerca en Sanitat Animal (CReSA), Campus de la Universitat Autònoma de Barcelona (UAB), Bellaterra, Spain; 2 IRTA Programa de Sanitat Animal, Centre de Recerca en Sanitat Animal (CReSA), Campus de la UAB, Bellaterra, Spain; 3 Infectious Disease Service, Germans Trias i Pujol Research Institute and Hospital, Badalona, Spain; 4 IrsiCaixa AIDS Research Institute, Hospital Germans Trias i Pujol, Institute for Health Science Research Germans Trias i Pujol (IGTP), Badalona, Spain; 5 University of Vic-Central University of Catalonia (UVic-UCC), Vic, Spain; 6 CIBERINFEC, Centro de Investigación Biomédica en Red, Instituto de Salud Carlos III, Madrid, Spain; 7 ICREA, Barcelona, Spain; 8 Department de Sanitat i Anatomia Animals, Facultat de Veterinària, Campus de la UAB, Bellaterra, Spain

**Keywords:** Central nervous system infections, Viral infection

## Abstract

More than 40% of individuals infected by severe acute respiratory syndrome coronavirus 2 (SARS-CoV-2) have experienced persistent or relapsing multi-systemic symptoms months after the onset of coronavirus disease 2019 (COVID-19). This post-COVID-19 condition (PCC) has debilitating effects on the daily life of patients and encompasses a broad spectrum of neurological and neuropsychiatric symptoms including olfactory and gustative impairment, difficulty with concentration and short-term memory, sleep disorders and depression. Animal models have been instrumental to understand acute COVID-19 and validate prophylactic and therapeutic interventions. Similarly, studies post-viral clearance in hamsters, mice and nonhuman primates inoculated with SARS-CoV-2 have been useful to unveil some of the aspects of PCC. Transcriptomic alterations in the central nervous system, persistent activation of immune cells and impaired hippocampal neurogenesis seem to have a critical role in the neurological manifestations observed in animal models infected with SARS-CoV-2. Interestingly, the proinflammatory transcriptomic profile observed in the central nervous system of SARS-CoV-2-inoculated mice partially overlaps with the pathological changes that affect microglia in humans during Alzheimer’s disease and aging, suggesting shared mechanisms between these conditions. None of the currently available animal models fully replicates PCC in humans; therefore, multiple models, together with the fine-tuning of experimental conditions, will probably be needed to understand the mechanisms of PCC neurological symptoms. Moreover, given that the intrinsic characteristics of the new variants of concern and the immunological status of individuals might influence PCC manifestations, more studies are needed to explore the role of these factors and their combinations in PCC, adding further complexity to the design of experimental models.

## Abstract (web summary)

In this Perspective, the authors summarize the current knowledge on the post-COVID-19 condition, focusing on the neurological manifestations, and discuss the applicability of existing animal models to recapitulate human condition.

## Main

The severe acute respiratory syndrome coronavirus 2 (SARS-CoV-2) is a highly pathogenic betacoronavirus that emerged in China in late 2019; its rapid spread across the world resulted in the coronavirus disease 2019 (COVID-19) pandemic^1,2^. SARS-CoV-2 shares its genomic organization with other betacoronaviruses, encoding four structural proteins (Spike, Envelope, Membrane and Nucleoprotein), and several nonstructural proteins. The structural proteins of SARS-CoV-2 share more than 90% amino acid identity with SARS-CoV, except for the Spike (S) protein, which displays an insertion of a polybasic furin cleavage site at the junction between the S1 and S2 subunits of the receptor-binding domain (RBD) of SARS-CoV-2 (ref. ^3^). Antibodies against the RBD have been detected in the majority of patients with COVID-19, often with neutralizing activity, prompting the early development of RBD and S-based vaccines^4^.

Since the early phases of the pandemic, it became evident that the novel disease was a multi-systemic disorder. Besides the respiratory outcome, patients affected by COVID-19 may suffer from extrapulmonary manifestations, including hematological, cardiovascular, gastrointestinal, dermatological, endocrine, ophthalmological and neurological symptoms. Initially, it was unclear whether all these clinical–pathological features resulted from a widespread replication of the virus itself or a consequence of the virus-induced inflammatory and immunological responses^5–7^. Nowadays, both hypotheses are considered valid^8–10^.

Owing to the virtually ubiquitous expression of angiotensin-converting enzyme 2 (ACE2), the main cell receptor for SARS-CoV-2 that is expressed on endothelial and smooth muscle cells, it was suspected that the multi-organ effects of SARS-CoV-2 infection were directly linked to viral presence and replication^11^. However, the mechanisms underlying SARS-CoV-2 multi-organ spread are still unclear because viremia is not a usual outcome of the infection and has been detected in very few severe cases^12–14^. As of May 2023, only one severe case with high and persistent SARS-CoV-2 viremia leading to meningitis, probably facilitated by blood–brain barrier (BBB) injury, has been well documented^15^.

**Fig. 1:**
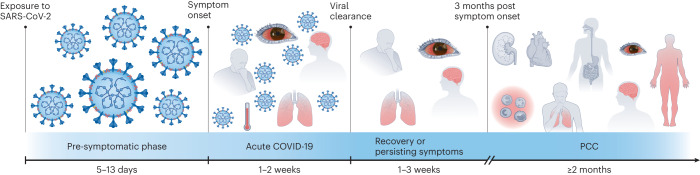
Timeline of acute COVID-19 and PCC in humans. The timings stated in each section of the figure indicate the duration of the corresponding phase of the disease. After exposure to SARS-CoV-2, a pre-symptomatic phase characterized by viral replication and early viral shedding precedes the onset of symptoms. The most common symptoms of acute COVID-19 are fever, cough, headache, dyspnea and fatigue; during this phase, the virus is still replicating, and the patient is contagious. In the weeks immediately after viral clearance, symptoms can either remit or persist. The common consensus for PCC is the persistence or new onset of symptoms three months after the beginning of the acute phase, which lasts for at least two months^1,19,95–97^. Adapted from ref. ^82^, CC BY 4.0 (https://creativecommons.org/licenses/by/4.0/).

As soon as the very first wave of COVID-19 in 2020 waned, it was observed that a proportion of patients were experiencing symptoms weeks or months after viral clearance. The most common post-acute symptoms reported were shortness of breath, fatigue, pain, increased heart rate, anosmia–dysgeusia, headache and cognitive and neurological impairment, which can considerably impact daily activities and quality of life^16–18^. This condition initially named ‘long-COVID’, and now known as ‘post-COVID-19 condition’ (PCC), was clinically defined by a Delphi consensus process in 2022 as the presence of symptoms in individuals with a probable or confirmed SARS-CoV-2 infection, 3 months from the onset of COVID-19. Such symptoms may occur after recovery from acute COVID-19 or persist from the initial disease and may fluctuate or relapse over time. Such a definition might be different for children^19^. Differences between cohorts reported in the literature also suggest the existence of two populations: (1) patients with symptoms that persist after the resolution of a severe acute infection, and (2) patients with symptoms that develop only in the post-acute phase of the disease, usually after mild COVID-19 (ref. ^20^). PCC is also referred to as post-acute sequelae of COVID-19. A timeline of acute COVID-19 and PCC is represented in Fig. 1.

PCC has a debilitating effect on the daily life of patients, reducing their ability to work or engage in social and physical activities. Given the hundreds of millions of individuals who have become infected with SARS-CoV-2, the social implications of the long-term consequences of COVID-19 are of great relevance and add to the health burden caused by the pandemic. The societal and public health impact of PCC is even more concerning given that PCC has also been linked to an increased incidence of noncommunicable diseases. However, the mechanisms of PCC pathogenesis are far from being satisfactorily unveiled, leaving patients suffering from this condition without proper treatment.

Animal models have been instrumental in the understanding of the acute phase of SARS-CoV-2 infection; the development of adequate models allowing the long-term follow-up of convalescent animals will be useful to better understand PCC pathology and to design counteracting measures. Among the several species used as animal models of SARS-CoV-2 infection^21^, some have proven to be suitable for studying various aspects of PCC. However, due to the differences in lifespan and viral kinetics between humans and most laboratory animals, translating the PCC concept to other species can be challenging. In general, it is considered that all animal studies extending beyond viral clearance and displaying multi-organ damage could provide important information about the long-term effects of SARS-CoV-2 infection and their mechanisms. Golden Syrian hamsters (GSH), mice (including transgenic or transduced with human ACE2 (hACE2)) and nonhuman primates (NHP) have all been used for the study of the long-term neurological sequelae of SARS-CoV-2 infection.

This Perspective aims to summarize the current knowledge on PCC, with a particular focus on its neurological manifestations, and to discuss the applicability of existing animal models to recapitulate some of the symptoms observed in patients with PCC.

## Potential mechanisms of PCC

It is estimated that, worldwide, 43% of patients who had SARS-CoV-2 infection have experienced PCC, with regional prevalence ranging from 11.5% to 75.0% (refs. ^22–26^). A fraction of patients who survived severe acute respiratory syndrome (SARS, caused by SARS-CoV) and Middle East respiratory syndrome (MERS, caused by MERS-CoV) also experienced psychological sequelae and chronic fatigue after the resolution of the infection, but PCC seems to be more pleiotropic than post-SARS and post-MERS syndromes^27,28^. Whether this is actually the case, or whether this assumption is skewed by the higher number of people infected by SARS-CoV-2, remains to be determined. Factors such as the severity of acute COVID-19, as well as comorbidities or specific demographic characteristics (respiratory disease, body mass index, older age and Black or Asian ethnicity), have been associated with symptom persistence and intensity^29^. Nonetheless, PCC has been reported even in patients who had mild to moderate acute disease^30^. Interestingly, PCC seems to be more frequently associated with the female gender, while studies have shown that severe acute disease is more prevalent in male patients^30–33^.

**Fig. 2:**
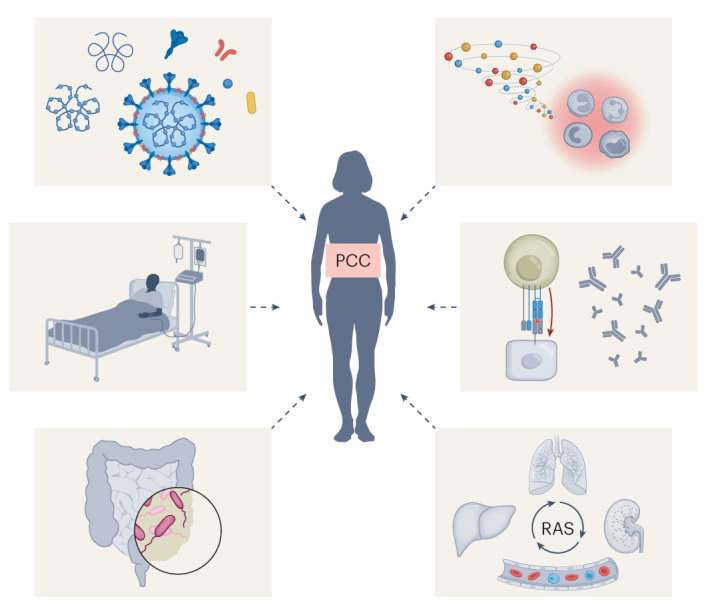
Putative mechanisms of PCC. From top left in clockwise order: persistence of the whole virus or viral fragments; prolonged inflammation and immunological aberrations; virally induced autoimmunity; alterations of the RAS; microbiome dysbiosis; post-intensive care syndrome.

The mechanisms probably contributing to the pathogenesis of PCC fall under five main categories (Fig. 2): (1) persistence of the whole virus or viral fragments; (2) prolonged inflammation and immunological aberrations; (3) virally induced autoimmunity; (4) alterations of the renin–angiotensin system (RAS); and/or (5) microbiome dysbiosis^29,34–36^. In addition to the aforementioned mechanisms, post-intensive care syndrome (a condition of impaired physical, cognitive and psychiatric state, irrespective of the critical illness suffered by the patient) is also thought to contribute to PCC in patients with severe COVID-19 who had required intensive care unit hospitalization^29,37^. Similar to acute COVID-19, PCC encompasses extrapulmonary symptoms such as hematological and immune-related, cardiovascular, renal, gastrointestinal and hepatobiliary, endocrine, neurological, ophthalmological and dermatological disorders summarized in Table 1 (refs. ^29,34,35,38^). In this Perspective, we will focus solely on the neurological symptoms of PCC.

**Table 1:** Most common manifestations of both acute and persistent COVID-19 in humans

Organ or system	Lesions and symptoms
Respiratory	• Dyspnea • Decreased exercise capacity • Chronic cough • Exacerbation of asthma
Cardiovascular	• Palpitation • Chest pain and tightness • Arrhythmias • Postural orthostatic tachycardia • Orthostatic hypotension • Vasovagal syncope • Myocarditis and/or pericarditis • Myocardial ischemia • Ventricular dysfunction • Venous thromboembolic events
Cerebrovascular	• Ischemic stroke
Renal	• Impaired renal function • Severe acute kidney injury • Microhematuria
Endocrine	• New onset or worsening of existing diabetes mellitus • Glycemic abnormalities • Diabetic ketoacidosis • Hormonal abnormalities • Subacute thyroiditis • Autoimmune thyroiditis • Bone demineralization • Lipid abnormalities • Hot and cold sensation
Gastrointestinal and hepatobiliary	• Abdominal pain • Nausea • Loss of appetite • Altered bowel motility • Irritable bowel syndrome • Dysphagia • Hepatitis
Dermatological	• Hair loss • Skin rash • Nail alterations • Papulo-squamous eruptions (pernio- or chilblain-like)
Musculoskeletal	• Myalgia • Arthralgia • Asthenia
Neuropsychiatric	• Migraine-like headache • Olfactory and gustatory impairments • Difficulties with concentration and short-term memory • Dizziness • Imbalance • Encephalitis • Myelopathy • Neuropathy • Depressive symptoms • Anxiety • Insomnia and sleep disorders • Mood disorders • Post-traumatic stress disorder
Miscellaneous	• Fever • Hearing loss • Tinnitus • Red eyes • Sore throat • Light and sound sensitivity • Blurry vision • Excessive bruising
Multi-system inflammatory syndrome in children (MIS-C)	• Fever • Abdominal pain • Vomiting • Diarrhea • Skin rash • Mucocutaneous lesions • Hypotension • Cardiovascular and neurological symptoms

The information listed in this table was extracted from refs. ^29,34,35,38^.

## Neurological symptoms of PCC

Approximately one-third of patients with acute COVID-19 report neurological symptoms including headache, dizziness, anosmia, ageusia, anorexia, myalgia and fatigue; more severe manifestations such as stroke, encephalopathy (with or without encephalitis) and Guillain–Barré syndrome have also been described^5,39^. Considering the broad spectrum of neurological manifestations of PCC, different mechanisms may be involved in the pathogenesis of specific disorders. ‘Brain fog’, which is one of the most common self-reported symptoms in both acute and post-acute COVID-19, describes a feeling of being mentally slow (bradyphrenia), with confusion, memory loss and difficulty concentrating^40^. This phenomenon is very similar to the so-called chemo-brain, often experienced by patients with cancer during or after chemotherapy; this condition also known as cancer-related or chemotherapy-related cognitive impairment is believed to be caused by neuroinflammation^41,42^.

So far, evidence of SARS-CoV-2 infection in the central nervous system (CNS) is limited to post-mortem samples from severe cases; CNS SARS-CoV-2 infection is also supported by transcriptomic data reporting ACE2 expression in both neuronal and nonneuronal cells of the human brain^43,44^. Although not confirmed in humans or experimental animal models following SARS-CoV-2 natural infection, studies have shown that the soluble monomeric S1 subunit of the Spike protein can cross the mouse BBB in vivo after both intravenous and intranasal administration, and spread to different areas of the brain^45^. Given that coronavirus S1 subunits are often shed from the virions by the action of host proteases located on cell surface, it cannot be ruled out that soluble SARS-CoV-2 Spike S1 triggers inflammation and tissue damage, even in the absence of intact viral particles.

On the other hand, the presence of the virus itself has seldom been demonstrated in the cerebrospinal fluid (CSF) of patients; an altered CSF cytokine profile (with increased levels of IL-6, IL-8 and IL-10) has also been reported in one case of SARS-CoV-2-associated encephalitis^46–48^. Furthermore, a sizable single-cell transcriptomic study analyzing the brains of patients who succumbed to COVID-19 did not find any molecular traces of SARS-CoV-2 in the brain, but did identify the upregulation of inflammation-related genes (predominantly in astrocytes), antiviral defense markers (interferon-induced transmembrane protein 3 among others) and T cell infiltration^49^. The transcriptomic perturbations identified in the study, in particular those suggesting compromised neurotransmission and information processing, share features with pathological cell states of neurodegenerative disease. Moreover, Alzheimer’s-like features such as excessive levels of reactive oxygen species (ROS), activation of the TGF-β pathway and increased tau phosphorylation have been found in the brains of these patients. It is interesting to note that olfactory dysfunctions are also common symptoms of early Alzheimer’s and Parkinson’s diseases, further suggesting a shared mechanism between PCC and neurodegenerative diseases^50,51^. Table 2 compares the hallmark neurological symptoms and the associated lesions and study findings between PCC and these neurodegenerative diseases.

**Table 2:** Shared neurological symptoms and lesions between PCC, Parkinson’s and Alzheimer’s diseases

	Condition		
	PCC	Parkinson’s disease	Alzheimer’s disease
**Symptoms**			
Headache	✓	**–**	**–**
Dizziness	✓	**–**	**–**
Anosmia	✓	✓	✓
Ageusia	✓	**–**	**–**
Anorexia	✓	**–**	**–**
Myalgia	✓	✓	**–**
Fatigue	✓	**–**	**–**
Bradyphrenia	✓	**–**	✓
Confusion	✓	**–**	✓
Memory loss	✓	**–**	✓
Impaired concentration	✓	**–**	✓
Sleep disorders	✓	✓	**–**
Depression	✓	✓	**–**
**Lesions and study findings**			
Altered CSF cytokine profile	✓	✓	✓
Microglia activation	✓	**–**	✓
Astrocyte activation	✓	**–**	✓
T cell infiltration	✓	**–**	**–**
Excessive ROS	✓	✓	✓
Activated TGF-β pathway	✓	**–**	✓
Tau hyper-phosphorylation	✓	**–**	✓
α-Synuclein hyper-phosphorylation	✓	✓	✓

The information listed in this table was extracted from refs. ^5,39,40,46,47,49–51,61,92,93^.

## Animal models for the study of PCC

In this Perspective, we considered all studies that followed animals for more than 14 days post-inoculation (dpi) as ‘long-term studies’.

### GSH

The GSH (*Mesocricetus auratus*) is a valuable small animal model for the study of SARS-CoV-2 infection, because the GSH is naturally susceptible to infection^52^. A study has reported multi-organ damage with infiltration of inflammatory cells and necrosis in upper and lower airways, secondary lymphoid organs, the digestive tract, kidneys, adrenal gland and ovaries in this model 5–7 dpi with the SARS-CoV-2/WH-09/human/2020/CHN isolate. These lesions were very mild in nonrespiratory tissues and could still be present by 18 dpi. In addition, different levels of viral genomic RNA (detected by quantitative PCR with reverse transcription (RT–qPCR) and in situ hybridization) and/or S and N proteins (detected by immunohistochemistry) were detectable up to 7 dpi in nasopharynges, trachea, lungs, kidneys, testis, vesicular gland, prostate, adrenal gland, spleen and lymph nodes^53^. Similarly, another study showed that microglial activation and accumulation of hyperphosphorylated tau and α-synuclein persisted in the brain of SARS-CoV-2-inoculated hamsters after viral clearance (14 dpi), in the olfactory bulb (OB) and in the cortical neurons, respectively, without any evidence of neuroinvasion (as shown by a lack of detectable S protein by immunohistochemistry), which suggests these pathological features contribute to the long-lasting neurological signs of SARS-CoV-2 infection^54^.

Additionally, a long-term study compared the systemic effects of SARS-CoV-2 (USA-WA1/2020 isolate) with those of influenza A virus (IAV) pandemic strain H1N1 (A/California/04/2009 isolate) in GSH for up to 31 dpi (a timepoint when infectious particles for either infection were no longer detectable in any of the investigated tissues)^55^. This study found that the histological alterations induced by SARS-CoV-2 in the lungs (peribronchiolar metaplasia and monocyte and neutrophil infiltration in the alveolar space) and kidneys (areas of tubular atrophy and presence of proteinaceous fluid in the interstitial space) were more severe than those induced by IAV and persisted for a longer period after the resolution of infection. Moreover, the transcriptomic analysis of the olfactory tissue and of different areas of the CNS revealed that, while both viruses caused a similar pattern of perturbations in the CNS that persisted for over a month, SARS-CoV-2 induced a unique profile in the olfactory tissue of GSH. Thirty-one days after SARS-CoV-2 infection, genes associated with microglial activation and macrophage infiltration (these increases in gene expression were confirmed by immunostaining), T cell recruitment and activation, type I interferon and chemokine response were upregulated, while genes involved in sensory perception and olfactory capabilities were downregulated. At 26 dpi, SARS-CoV-2-infected GSH also showed a reduction in burying activity when subjected to the marble-burying assay compared with IAV-infected and noninfected hamsters, demonstrating a change in this spontaneous rodent behavior following SARS-CoV-2 infection^55^. Interestingly, injury and chronic inflammation of the OB have been previously linked to neurobehavioral disorders because OB function can impact sensory, emotional and cognitive processes given the proximity of the OB to the limbic system responsible for such reactions^56^. Therefore, the behavioral changes observed in SARS-CoV-2-infected GSH suggest that long-term OB inflammation causes neurodegenerative changes that are compatible with PCC neurological symptoms. This observation is in line with evidence of gray matter loss in the limbic and olfactory cortical areas of patients who have recovered from COVID-19, even with mild acute manifestations^55,57^.

In addition, SARS-CoV-2 infection in GSH has been linked to mechanical hypersensitivity during both the acute and post-acute phases (28 dpi) of SARS-CoV-2 infection, which resembles the somatosensory abnormalities observed in patients with PCC. This prolonged hypersensitivity coincided with the differential expression of several genes associated with neuro-oncological and neurodegenerative disorders (including glioblastoma, Alzheimer’s and Parkinson’s diseases and neurilemmoma) compared with noninfected hamsters. The same transcriptomic analysis predicted the contribution of macrophages and Schwann cells, already known to be involved in neuropathic pain and hypersensitivity, to the SARS-CoV-2-induced proinflammatory state. Interestingly, these results were obtained long after viral clearance, suggesting that the presence of viral transcript (but not infectious particles) and type I interferon response in the peripheral nervous system (dorsal root ganglia and spinal cord) during the first 24 h post-inoculation was sufficient to induce persistent maladaptive neuronal responses^58^.

### Mice

Given the substantial differences in amino acid sequences between human and mouse ACE2, the latter is not a functional receptor for the ancestral SARS-CoV-2 strain. hACE2-transgenic (hACE2-tg) mice, transient hACE2 expression and virus adaptation to the mouse are among the strategies employed to circumvent this limitation^21^. In addition, different humanized mice have been used as models of PCC, with a particular focus on the lungs and the respiratory tract. MISTRG6 humanized mice, reconstituted with human immune cells and transiently expressing hACE2 via an adeno-associated vector (AAV-hACE2), have been used to reproduce the human innate and adaptive responses to SARS-CoV-2 infection. Results from this model have suggested that human immune cells contribute to the pathological inflammatory response in the lungs and to viral RNA persistence, which was detected until 35 dpi, either as a result of direct infection of ACE2-expressing myeloid cells or as a residue of phagocyted infected epithelial cells^59^. Similar results have been obtained in DRAGA (HLA-A2.HLA-DR4.Rag1KO.IL2RγcKO.NOD) humanized mice, which when infused with human hematopoietic stem cells from cord blood reconstitute both a functional human immune system and hACE2-expressing human epithelial and endothelial cells in the lung and upper respiratory airways. Inoculated animals were followed for 25 days, which revealed mild to severe lung pathology with inflammatory infiltrate and alveolar damage until the end of the study^60^.

A longer-term study monitored neuroinflammation up to 7 weeks post-infection (wpi) with SARS-CoV-2 (isolate USA-WA1/2020) in wild-type CD1 mice transduced with AAV-hACE2 in the trachea and lungs by intratracheal injection^61^. This model simulates a mild respiratory infection with no to moderate lung pathology and no viral neuroinvasion. Increased protein levels of proinflammatory cytokines (IFNγ, IL-6, TNF, CXCL10, CCL7, CCL2, CCL11, BAFF and GMCSF) were observed both in the serum and CSF of infected mice as early as 7 dpi. Interestingly, CSF levels of C–C motif chemokine 11 (CCL11), known to limit neurogenesis and contribute to cognitive impairment, further increased until at least 7 wpi, while they normalized in the serum at the same timepoint. This increase in CCL11 CSF levels was associated with increased microglial activation in the subcortical white matter (as indicated by increased expression of allograft inflammatory factor 1 (AIF1; also known as IBA1) marker in CD68^+^ cells), which persisted during the entire study period. Moreover, transcriptomic analysis at 7 dpi and 7 wpi revealed an increase in the abundance of a chemokine-expressing subpopulation of the microglia as well as an altered profile in the homeostatic microglia subpopulation, with upregulation of genes associated with cytotoxicity, inflammation, antigen processing and presentation. Interestingly, this gene signature partially overlaps with the expression profiles of pathological cell states such as disease-associated microglia observed in Alzheimer’s disease, and white-matter-associated microglia observed in aging. SARS-CoV-2-infected mice also exhibited a persistent decrease in neurogenesis in the hippocampus and a decrease in myelinated axon density during the entire study period, as well as a decrease in mature oligodendrocytes (ASPA^+^ CC1^+^ population) at 7 dpi, which recovered by 7 wpi. The magnitude of myelin loss was strikingly similar to that observed after chemotherapy exposure, confirming the similarity between ‘COVID-19 brain fog’ and ‘chemo fog’^61^.

Studies using a mouse-adapted SARS-CoV-2 strain (MA10) have reported productive infection with severe lung injury and mortality in standard laboratory mice^62,63^. Although no virus was detected in the brain of intranasally infected animals, this model showed an upregulation of proinflammatory cytokine messenger RNAs, altered BBB permeability and an increase in IBA1^+^ microglial cells in the cortical region of 1-year-old *Rag*^*–/–*^ mice (a mouse model lacking mature lymphocytes). Histological analysis of aged *Rag*^*–/–*^ brain samples revealed prominent lymphocyte perivascular cuffing, indicating inflammation of blood vessels at 30-days post inoculation^64^. Long-term studies using the mouse adapted MA10 strain in immunocompetent mice are needed to confirm these findings and to determine the extent of brain damage and neurological impairment caused by SARS-CoV-2.

### NHPs

NHPs have been successfully used for the study of human brain disorders, viral infections and respiratory diseases, taking advantage of their phylogenetic proximity and anatomical and physiological similarity to humans^65–67^. Over the past 3 years, NHPs have been of great interest in the study of both acute and post-acute SARS-CoV-2 infection^21,68^.

A study following rhesus macaques and African green monkeys for 28 days after infection with the SARS-CoV-2 USA-WA1/2020 virus strain revealed neuroinflammation, as well as brain hypoxia, microhemorrhages and lesions similar to autopsy findings from patients who died after SARS-CoV-2 infection^69^. Neuroinflammation signatures, determined by the expression of the pan-microglial marker IBA1 and glial fibrillary acidic protein (GFAP), were greater in infected animals than in mock-infected control animals, and revealed more pronounced astrocytic hypertrophy in these animals. In addition, the inoculated animals in this study displayed neuronal morphological changes in the cerebellum and the brainstem, such as degenerated Purkinje neurons exhibiting cellular blebs and cytoplasmic vacuoles, and adjacent pyknotic glial cells with condensed nuclei. These lesions were found concomitantly with cleaved caspase 3 staining, indicating that apoptosis was one of the mechanisms underlying such neuronal degeneration. Very low levels of viral S RNA were detected in the brain of some of the infected animals; S expression was always limited to vascular endothelial cells, with no involvement of parenchymal cells. The virus was not detected in the CSF of infected NHPs, which might suggest hematological dissemination of the virus to the brain. Interestingly, hypoxic–ischemic injuries, with areas of intense positivity for the hypoxia-inducible factor 1α (HIF1α) were detected within and around blood vessels in the brain of infected animals^69^. Given that the expression of this protein can be upregulated by proinflammatory cytokines such as TNF — a factor abundant in the serum of patients with severe COVID-19 — and by the SARS-CoV-2 protein translated from ORF3 (ref. ^10^), at least in vitro, hypoxia could be both a direct and an indirect effect of SARS-CoV-2 infection. It is worth mentioning that signs of hypoxia were also detected in animals that did not develop severe respiratory disease, suggesting that it could be one of the causes of neurological disorder even after mild COVID-19, as observed in patients^70,71^.

The advanced age (13–21 years) of the NHPs used for this study might have acted as a confounding factor: all morphological changes and inflammatory features were also observed in mock-infected animals, although at a lower level. It is therefore probable that the infection contributed to the worsening of pre-existing, age-related lesions rather than being their triggering cause. Moreover, none of the 12 infected animals displayed clinical signs of neurological disorders, except for one female African green monkey that was found recumbent and marginally responsive to stimuli at 8 dpi (ref. ^69^).

### Animal models with CNS involvement during the acute phase of COVID-19

Considering the small number of models currently available for the study of the long-term neurological manifestations of SARS-CoV-2 infection, it is worth considering all preclinical studies that have shown either neuroinvasion or neuroinflammation during the acute phase of the infection.

In one study, infectious viral particles were isolated from different areas of the brain of SARS-CoV-2-infected hamsters 4 dpi, indicating that the virus can reach and productively replicate in the CNS of this animal model. Viral particles were also detected in the same animals' olfactory neuroepithelium, along with infiltrating myeloid cells and upregulated expression of proinflammatory markers^72^.

On the other hand, K18-hACE2-tg mice — mice carrying the hACE2 sequence under the control of the cytokeratin-18 (K18) gene promoter — are susceptible to SARS-CoV-2 infection, develop severe lesions including massive brain invasion, neuronal death and neuroinflammation, and succumb by 5–7 dpi when given a dose of either 10^3^ TCID50 SARS-CoV-2 or 10^5^ plaque forming unit intranasally^73–75^. This model has been successfully used for testing vaccines and antivirals but does not properly replicate human COVID-19. However, a short-term study in 2022 has now shown that the administration of aerosolized SARS-CoV-2 to K18-hACE2 mice leads to efficient respiratory infection and anosmia without fatal neuroinvasion, even at the same dose^76^. Similarly, a study showed that K18-hACE2 mice intranasally inoculated with 10^3^ plaque forming unit of the BA.1 (Omicron) variant develop mild respiratory pathology without neuroinvasion up to 7 dpi (ref. ^77^). Notwithstanding the differences in ACE2 expression and in the severity of SARS-CoV-2-induced disease between K18-hACE2 mice and humans, it is possible to achieve a nonfatal disease via a fine modulation of the experimental inoculation (that is, use of a sublethal dose or a less pathological variant, and use of a more natural administration route such as aerosolization), which might extend the survival of the infected animals sufficiently to allow the study of long-term consequences of SARS-CoV-2 infection^78^.

Less extensively used than the K18-hACE2 mice, hACE2 knock-in (KI) mice are considered a more physiologically relevant model, because the expression of the human receptor is under the control of the endogenous promoter of mACE2, thereby preventing ectopic expression. One short-term study using hACE2-KI mice has shown very low levels of SARS-CoV-2 RNA in the brain and OB of some of the inoculated mice at 7 dpi (ref. ^79^). The presence of replicating particles and the long-term effect of viral presence in the brain have not been investigated in these animals.

An early study on rhesus macaques infected with SARS-CoV-2 (isolate KMS1/2020) also showed neuroinvasion. Viral RNA was detected by RT–qPCR in the brain and spinal cord of the NHPs on day 9 post-intranasal infection, while it was detected as soon as 2 dpi and during the entire study period in the lungs and trachea. The animals in the study had transient viremia, suggesting systemic spread of the virus through the bloodstream. Histological analysis of the CNS also revealed hyperemia and edema in some of the samples^80^, but those findings might not be specific to SARS-CoV-2 infection.

Interestingly, ferrets (*Mustela putorius furo)* are susceptible to SARS-CoV-2 infection, but they develop only minor clinical signs even in the presence of mild histological lesions in the lower respiratory tract. In a study aimed at determining the effect of age in SARS-CoV-2-induced pathology and immunity, viral RNA was detected in extra respiratory tissues including the brain of both young and adult animals up to 5 dpi, but the production of infectious virus was not demonstrated^81^. Moreover, no evidence of neurological clinical signs was observed.

## Conclusion and future perspectives

Currently available animal models for SARS-CoV-2 infection should be considered useful tools for understanding the long-term consequences of SARS-CoV-2 infection^82^ (Table 3). In particular, studies in hamster and mouse models have confirmed that some of the neurological manifestations of PCC share molecular mechanisms with neurodegenerative disease characterized by neuroinflammation and proteinopathy^53,54,61^. Promising results have also been obtained from long-term studies in NHPs, which share similar infection kinetics with humans. However, data on neurological signs in NHPs following SARS-CoV-2 infection are still scarce, and more studies are needed to determine the actual usefulness of this model^69^. On the other hand, ferrets seem not to be an adequate model for the study of the long-term consequences of SARS-CoV-2 infection, because they do not develop any clinical signs, and active viral replication has not been clearly demonstrated in lung samples^81^. Given that none of the models presented in this Perspective seems to comprehensively phenocopy PCC in humans, it is reasonable to foresee that multiple animal models will be necessary to unveil the several aspects of this multi-systemic condition.

**Table 3:** List of potential PCC animal models

Model	Similarities to human disease	Differences with the human disease	Neurological manifestations and laboratory findings	Technical considerations	References
GSH	• Naturally susceptible to SARS-CoV-2 infection • Multi-organ damage • No evidence of neuroinvasion • Spontaneous viral clearance	• Rapid humoral neutralizing immune response • Severe disease manifestations are limited by quick spontaneous viral clearance	• Accumulation of hyper -phosphorylated tau and α-synuclein in cortical neurons • Transcriptomic perturbations in the CNS • Behavioral changes	• Easy handling in the laboratory • Lack of hamster-specific research tools for the study of immune activation	^21,52–55,72,86^
Wild-type laboratory mice	• Susceptible to the Alpha, Beta, and Omicron SARS-CoV-2 variants with mild disease course	• Not susceptible to SARS-CoV-2 ancestral strain infection	• Neuroinflammation (observed with SARS-CoV-2 mouse adapted strain; studies limited to the acute phase)	• Easy handling in the laboratory • Wide availability of research tools	^21,64,84–86^
K18-hACE2-tg mice	• Susceptible to SARS-CoV-2 infection (multiple variants tested)	• Massive neuroinvasion and neuroinflammation • Lethal infection without viral clearance by 7 dpi	• Nonsuppurative meningoencephalitis • Diffuse astrogliosis and microgliosis • Apathy, depression, trembling and seizures	• Easy handling in the laboratory • Wide availability of research tools	^21,43,73,74,76,78^
hACE2-KI mice	• Susceptible to SARS-CoV-2 infection	• Minimal lung pathology	• Low amounts of viral RNA occasionally detected in brain samples • Studies limited to the acute phase	• Easy handling in the laboratory • Wide availability of research tools	^78,94^
AAV-hACE2 transduced mice (multiple strains)	• Mild to moderate respiratory disease • No neuroinvasion	• Targeted expression of hACE2 determines which organs and tissues are infected by SARS-CoV-2 • Transient hACE2 expression, dependent on the turnover rate of the transduced cells	• Increased CSF levels of CCL11 • Increased microglial activation in the subcortical white matter • Decreased neurogenesis in the hippocampus • Myelin loss	• Variability in results might depend on the AAV batch. • If used in different mouse strains (such as different humanized mice), the model allows for the study of specific pathways or immune cell types	^59,61^
NHPs	• Naturally susceptible to SARS-CoV-2 infection • Neuroinflammation observed without evidence of neuroinvasion • Spontaneous viral clearance	• Different NHP species display a heterogeneous spectrum of COVID-19 symptoms	• Neuroinflammation • Brain hypoxia • Neuronal morphological changes in cerebellum and brainstem	• Ethical limitations • Handling in the laboratory may be difficult depending on the species of choice	^68,69,80^

AAV, adeno-associated virus.

When focusing on the neurological manifestations of PCC, it is important to consider that productive SARS-CoV-2 infection of the CNS might not be necessary for the development of cognitive and behavioral signs, because these manifestations might be triggered by inflammatory signals originating in other tissues. This is particularly relevant for K18-hACE2-tg mice, one of the most used small animal models for SARS-CoV-2 infection so far, which develop massive lethal neuroinvasion upon intranasal SARS-CoV-2 inoculation; this limitation has been overcome by the use of an aerosolized inoculum instead of direct intranasal inoculation^73,74,76^.

All studies published so far have analyzed the long-term consequences of SARS-CoV-2 infections in naïve animals using the ancestral strain. However, after the first wave of the COVID-19 pandemic, several variants of concern (VOCs) have appeared with different characteristics (such as transmissibility, replication abilities and lesion severity)^83^ that may influence the intensity and/or duration of possible PCC manifestations. In this regard, it is worth mentioning that the B.1.1.7 (Alpha), B.1.351 (Beta) and BA.1.1 (Omicron) variants can infect wild-type mice causing a mild disease without neurological signs during the acute phase^84–86^. This points to the possibility of developing mouse models to study the infection by specific VOCs of SARS-CoV-2, which would be extremely helpful for characterizing the long-term consequences of SARS-CoV-2 in people infected after the first wave of the pandemic. The use of such models might help to dissect the molecular pathways associated with different PCC symptoms when they are observed specifically with certain VOCs.

Moreover, the global COVID-19 vaccine rollout has reduced the percentage of the immunologically naïve population. An interesting point to consider is that PCC occurs also after vaccine breakthrough infections; whether PCC would then substantially differ from the condition observed in nonvaccinated naïve patients remains a question to be answered^87^. All these factors might limit the translatability of the currently available animal models to the entire affected population and call for the development of models exploring the influence of different vaccination status and VOCs on PCC; however, such models might require longer follow-up time and increase the complexity of the studies. Translating the neurological and cognitive symptoms experienced by patients with COVID-19 and PCC into objective and measurable outcomes for animal studies is a necessary step, which adds another level of complexity to the preclinical studies on PCC.

A practical aspect to be considered when carrying out long-term in vivo studies of SARS-CoV-2 infection is the requirement for biosafety level 3 animal facilities and adequately trained personnel. Pseudoviruses have been used to develop in vivo models of infection by highly pathogenic agents including SARS-CoV-2 (refs. ^88–90^), but to our knowledge, this technology has not been used yet to study the long-term effect of COVID-19. Pseudoviruses are a promising alternative to the use of infectious virus, which would also circumvent the biosafety level 3 requirements.

The heterogeneity of PCC manifestations observed in patients and the finding that PCC is apparently independent of the severity of the prior acute infection makes it unlikely that a single animal model will be able to fully recapitulate the human disease. This problem could be overcome by challenging the animals with a range of viral doses to induce a wider spectrum of clinical signs. In addition, animal models and humans do not always perfectly match in terms of genetic background, lifespan and aging processes, among other factors, which might affect responses to SARS-CoV-2. As already pointed out by other authors, a useful strategy to experimentally reproduce such variability would be to introduce systematic heterogeneity in the study design^91^.

Given that none of the currently available animal models seems to fully replicate PCC, it seems sensible to use multiple models to study different aspects of this condition. In particular, we think that GSHs are suitable for profiling neuroinflammation signature after SARS-CoV-2 inoculation, while mouse models would be the best tool to test sublethal doses of SARS-CoV-2 and less virulent VOCs as a mean to extend animal survival and observe the potential long-term effects of the infection.
